# Disease phenotype of classical sheep scrapie is changed upon experimental passage through white-tailed deer

**DOI:** 10.1371/journal.ppat.1011815

**Published:** 2023-12-04

**Authors:** Robyn D. Kokemuller, S. Jo Moore, Jifeng Bian, M. Heather West Greenlee, Justin J. Greenlee

**Affiliations:** 1 Virus and Prion Research Unit, National Animal Disease Center, USDA, ARS, Ames, Iowa, United States of America; 2 Department of Biomedical Sciences, Iowa State University College of Veterinary Medicine, Ames, Iowa, United States of America; Creighton University, UNITED STATES

## Abstract

Prion agents occur in strains that are encoded by the structure of the misfolded prion protein (PrP^Sc^). Prion strains can influence disease phenotype and the potential for interspecies transmission. Little is known about the potential transmission of prions between sheep and deer. Previously, the classical US scrapie isolate (No.13-7) had a 100% attack rate in white-tailed deer after oronasal challenge. The purpose of this study was to test the susceptibility of sheep to challenge with the scrapie agent after passage through white-tailed deer (WTD scrapie). Lambs of various prion protein genotypes were oronasally challenged with WTD scrapie. Sheep were euthanized and necropsied upon development of clinical signs or at the end of the experiment (72 months post-inoculation). Enzyme immunoassay, western blot, and immunohistochemistry demonstrated PrP^Sc^ in 4 of 10 sheep with the fastest incubation occurring in VRQ/VRQ sheep, which contrasts the original No.13-7 inoculum with a faster incubation in ARQ/ARQ sheep. Shorter incubation periods in VRQ/VRQ sheep than ARQ/ARQ sheep after passage through deer was suggestive of a phenotype change, so comparisons were made in ovinized mice and with sheep with known strains of classical sheep scrapie: No. 13–7 and x-124 (that has a more rapid incubation in VRQ/VRQ sheep). After mouse bioassay, the WTD scrapie and x-124 isolates have similar incubation periods and PrP^Sc^ conformational stability that are markedly different than the original No. 13–7 inoculum. Furthermore, brain tissues of sheep with WTD scrapie and x-124 scrapie have similar patterns of immunoreactivity that are distinct from sheep with No. 13–7 scrapie. Multiple lines of evidence suggest a phenotype switch when No. 13–7 scrapie prions are passaged through deer. This represents one example of interspecies transmission of prions resulting in the emergence or selection of new strain properties that could confound disease eradication and control efforts.

## Author summary

Passage of the sheep-derived US No. 13–7 classical scrapie isolate through white-tailed deer results in a change in disease phenotype that is observed when the deer-passaged scrapie agent is inoculated back into sheep or ovinized mice. Upon passage back to sheep, the relationship between incubation period and sheep *PRNP* genotype is reversed from the original inoculum. Whereas inoculation with the original No.13-7 scrapie agent results in a shorter incubation period in sheep with the ARQ/ARQ genotype as compared to VRQ/VRQ sheep, the deer-passaged scrapie agent results in a shorter incubation period in VRQ/VRQ sheep. In addition, passage of the No.13-7 isolate through deer results in a change in the pattern of PrP^Sc^ deposition in the brain of affected sheep. Taken together with the results of bioassay and conformational stability assays this work supports emergence of strain properties different from the No. 13–7 inoculum and consistent with another classical scrapie strain called x-124. Interspecies transmission of the classical scrapie agent can result in a phenotype switch through emergence of new scrapie strain properties that could potentially expand the potential host range.

## Introduction

Transmissible spongiform encephalopathies (TSEs) or prion diseases are neurodegenerative diseases that result from misfolding of the prion protein from the normal cellular form (PrP^C^) to the disease-associated form (PrP^Sc^) [1]. TSEs affect sheep (scrapie), deer (chronic wasting disease; CWD), cattle (bovine spongiform encephalopathy), and humans (Creutzfeldt-Jakob disease). There are multiple strains of prion agent that exhibit unique prion disease phenotypes (e.g. incubation period, location of PrP^Sc^ deposition, *PRNP* genotype of susceptible animals, conformational stability, electrophoretic mobility, reactivity to N-terminal antibodies after proteinase-K digestion) that may be encoded in the tertiary and/or quaternary structure of the misfolded protein [2]. Disease phenotype also can be influenced by genotype of the donor, and the species or tissue from which the TSE is derived [3].

Amino acid polymorphisms in the prion protein at codons 136, 154, and 171 can affect susceptibility and incubation periods after exposure to classical scrapie prions. Valine (V) 136, arginine (R) 154, and glutamine (Q) 171 (VRQ) is considered the most scrapie susceptible haplotype. Mild resistance to scrapie is associated with alanine (A) 136, histidine (H)154, and glutamine (Q) 171 (AHQ) [4]. The strongest resistance to the scrapie agent is associated with arginine (R) at codon 171 (ARR haplotype) [5]. PRNP genotypes can also modulate incubation periods of various scrapie strains. In previous studies, we have reported on two classical scrapie isolates: x-124 has a more rapid incubation period in VRQ/VRQ sheep, whereas No. 13–7 has a more rapid incubation period in ARQ/ARQ sheep [6]. Since it is possible for sheep and deer to share the same grazing lands, it is important to understand the potential interspecies transmission of the scrapie and CWD agents. To date, sheep oronasally exposed to the CWD agent have failed to develop clinical signs of prion disease with evidence of PrP^Sc^ in the lymph nodes and palatine tonsil of only a single sheep assessed at 60 months post-inoculation [7]. However, the attack rate in white-tailed deer intracranially or oronasally inoculated with the US scrapie isolate (No.13-7) was 100% in deer expressing either GS96 or SS96 *PRNP* [8,9]. The purpose of this study was to determine if sheep are susceptible to oronasal challenge with the scrapie agent from white-tailed deer.

## Results

The objective of this study was to determine if Suffolk sheep were susceptible to oronasal challenge with the scrapie agent derived from white-tailed deer (WTD scrapie). We used inoculum made from either cerebrum or brainstem from deer that developed disease after oronasal inoculation with the No.13-7 scrapie agent from sheep because these regions had two distinct western blot patterns. Cerebrum had a lower apparent molecular mass (scrapie-like) pattern while brainstem at the level of the obex had a higher migration (CWD-like) pattern [8,9].

Sheep results are summarized in Table 1. Three of the five sheep inoculated with the WTD scrapie agent from deer cerebrum developed clinical signs of scrapie including ataxia, slow to rise, biting or rubbing the flank area, weight loss, and hunched posture. The first two sheep to develop clinical signs at 28 and 31 MPI had the VRQ/VRQ genotype. One of two sheep with the ARQ/ARQ genotype developed disease at 48 MPI. The second ARQ/ARQ sheep (sheep 4) was euthanized at 56 months post-inoculation due to intercurrent disease (recurrent lameness). This is in contrast to the original No.13-7 isolate that had a faster incubation period in ARQ/ARQ sheep (20 MPI) than VRQ/VRQ sheep (27 MPI) [6] after oronasal exposure with 1 ml of a 10% (w/v) whole brain homogenate from an ARQ/ARQ sheep.

**Table 1:** PrP^Sc^ distribution in sheep inoculated with the scrapie agent derived from deer.

Animal Number	PRNP genotype	Inoculum	Months post-inoculation	Clinical Signs	LRS head	LRS other	Rectal	Retina	PNS
1	VRQ/VRQ	Cerebrum	31	+	+	+	+	+	+
2	VRQ/VRQ	Cerebrum	28	+	+	+	+	+	+
3	ARQ/ARQ	Cerebrum	48	+	+	+	+	-	+
4*	ARQ/ARQ	Cerebrum	56	-	-	-	-	-	-
5	VRQ/ARR	Cerebrum	72	-	-	-	-	-	-
6	VRQ/VRQ	Brainstem	65	+	+	-	+	+	+
7	VRQ/VRQ	Brainstem	72	-	-	-	-	-	-
8	ARQ/ARQ	Brainstem	72	-	-	-	-	-	-
9	ARQ/ARQ	Brainstem	72	-	-	-	-	-	-
10	VRQ/ARR	Obex	72	-	-	-	-	-	-

Animal data, survival time, presence of clinical signs at death, and PrP^Sc^ distribution for lymphoid tissues and non-brain nervous tissue. Sheep 4 was euthanized at 56 months-post inoculation due to intercurrent disease (recurring lameness).

Abbreviations: LRS: lymphoreticular system. PNS: peripheral nervous system tissue, CNS: central nervous system.

One sheep of two inoculated with the WTD scrapie agent from deer brainstem (genotype VRQ/VRQ) developed clinical signs at 65 MPI. Sheep challenged with the scrapie agent from either the cerebrum or brainstem from a scrapie-affected white-tailed deer had PrP^Sc^ accumulation in the CNS (including retina), lymphoid tissues, and peripheral nervous tissue (Table 1).

To determine if the relative amounts of PrP^Sc^ in the inoculum had an influence on the incubation period and attack rate of inoculated sheep, misfolded PrP present in each inoculum was tested by EIA. At equivalent dilutions, samples of deer cerebrum (shorter incubation period) had lower optical density values compared to samples of deer brainstem (S1 Table). Thus, the shorter incubation period in sheep inoculated with deer cerebrum was not due to more PrP^Sc^ in the inoculum.

**Fig 1:**
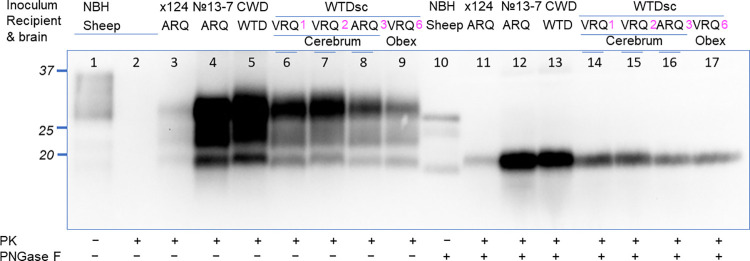
Western blot of sheep with clinical signs after inoculation with the WTD scrapie agent. Western blot comparison of sheep samples inoculated with WTD scrapie to the original No.13-7 classical scrapie inoculum, deer CWD, and x-124 classical scrapie. Monoclonal antibody 12B2 (epitope 93-WGQGG-97) were used for the detection of PrP^Sc^. Lane 1, normal sheep brain sample without PK treatment; lanes 2–9 samples of prion-diseased sheep treated with PK; lane 10, normal sheep brain sample treated with PNGase F; lanes 11–17 samples of prion-diseased sheep treated with PK and PNGase F. Sheep numbers 1, 2, 3 and 6 from the present study are indicated in magenta. Sheep inoculated with the WTD scrapie agent have a western blot pattern that is similar to deer CWD. This patterning was not dependent on sheep genotype or the brain region from deer with which they were inoculated. WTDsc, white-tailed deer inoculated with sheep classical scrapie No.13-7. The positions of molecular weight markers 37-, 25-, and 20-kDa are shown to the left of the blots.

Western blot analysis was used to determine the molecular profile of the sheep challenged with the WTD scrapie agent. Brain samples from sheep positive by immunohistochemistry (sheep 1–3 and 6) were also positive by western blot (Fig 1). Samples from sheep negative using immunohistochemistry (sheep 4, 5, and 7–10) were also negative on western blot. The western blot of brain material from all positive sheep had similar molecular profiles despite differences in inocula source and sheep genotype. Samples of brainstem at the level of the obex (referred to as obex throughout this manuscript) from sheep challenged with the WTD scrapie agent (Fig 1 lanes 6–9) had a molecular mass profile that was similar to sheep challenged with the original No.13-7 isolate (Fig 1, lane 4).

**Fig 2:**
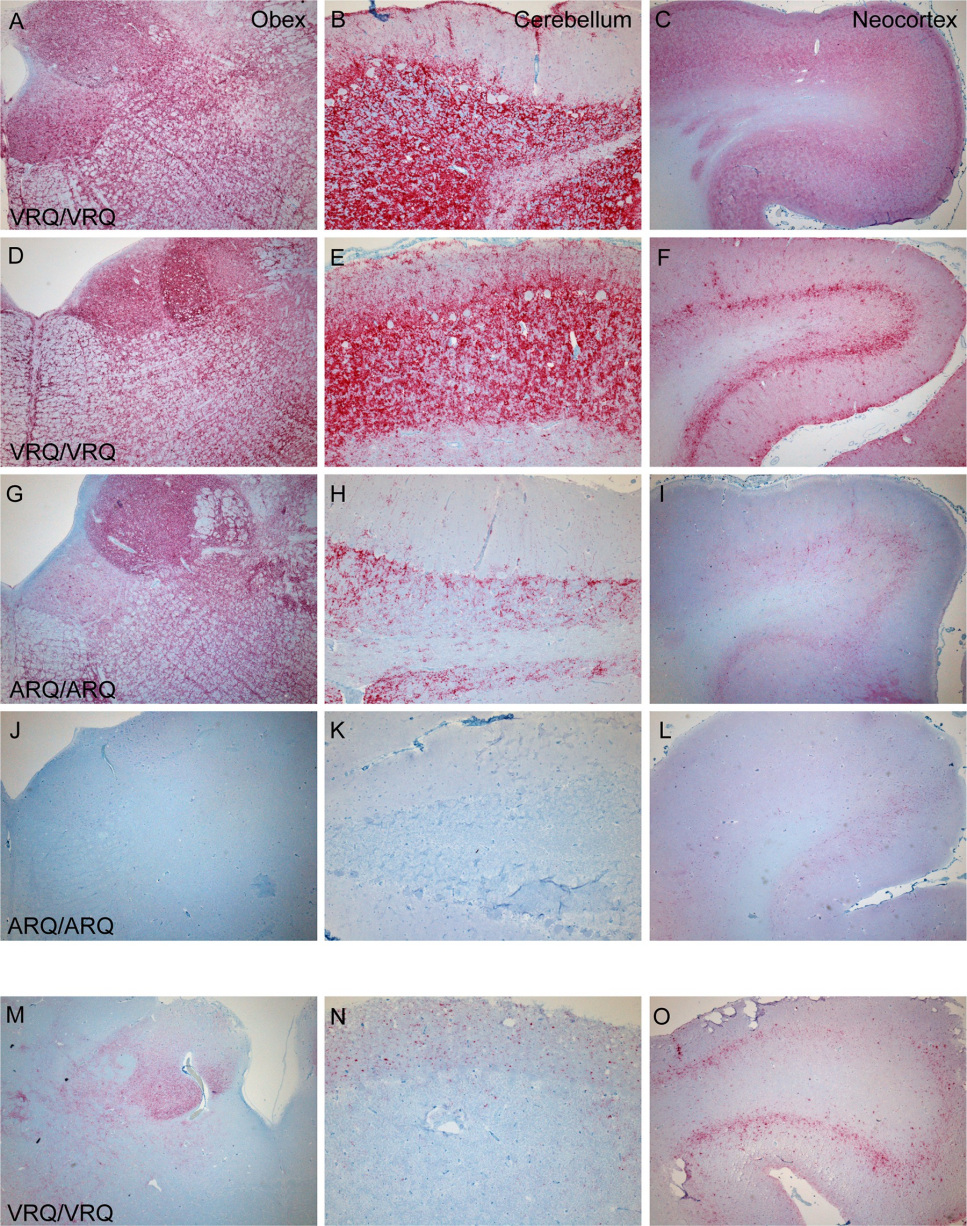
Comparison of PrP^Sc^ deposition between different sheep genotypes and inocula. PrP^Sc^ deposition in brain regions varies with sheep *PRNP* genotype and source of inoculum. Sheep inoculated with the WTD scrapie agent from cerebrum (sheep 1 (A-C), sheep 2 (D-F), sheep 3 (G-I), and sheep 4 (J-L)) are compared to a sheep inoculated with WTD scrapie agent from the obex (sheep 6 (M-O)). A VRQ/VRQ sheep inoculated with the WTD scrapie agent from cerebrum had intense widespread immunoreactivity in the brainstem at the level of the obex (A, D), cerebellum (B, E), and neocortex (C, F). An ARQ/ARQ sheep inoculated with the WTD scrapie agent from cerebrum had less intense but widespread accumulation in the obex (G), cerebellum (H), and neocortex (I). Representative brain regions of sheep 4 that was negative by EIA and WB lacks immunoreactivity (J-L). A VRQ/VRQ sheep inoculated with the WTD scrapie agent from obex (sheep 6) had mild PrP^Sc^ accumulation in the obex (M), cerebellum (N), and neocortex (O).

Potential differences in PrP^Sc^ distribution in sheep of different genotypes that received different inocula from white-tailed deer was assessed using immunohistochemistry with a monoclonal antibody against PrP. Sheep 1 and 2 (genotype VRQ/VRQ) inoculated with the WTD scrapie agent from cerebrum had intense, widespread immunoreactivity throughout the obex, cerebellum, and neocortex (Fig 2A and 2F). By comparison, sheep 3 (ARQ/ARQ) also inoculated with the scrapie agent from deer cerebrum had less intense, but widespread PrP^Sc^ immunoreactivity (Fig 2G and 2I). Sheep 4 (ARQ/ARQ) lacked immunoreactivity upon examination by IHC (Fig 2J and 2L). When compared to sheep inoculated with the WTD scrapie agent from deer cerebrum, sheep 6 (VRQ/VRQ) inoculated with the WTD scrapie agent from deer obex had less PrP^Sc^ immunoreactivity throughout the brain (Fig 2M and 2O). Also, in contrast to VRQ/VRQ sheep inoculated with the WTD scrapie agent from deer cerebrum (sheep 1 and 2, Fig 2E and 2B), the cerebellum of the VRQ/VRQ sheep inoculated with the WTD scrapie agent from deer obex (sheep 6, Fig 2N) had a majority of the PrP^Sc^ immunoreactivity in the cerebellar molecular layer and no immunoreactivity was detected in the white matter. These results demonstrate that there are differences in the intensity and distribution of immunoreactivity between sheep genotypes when challenged with the same inoculum and amongst sheep of the same genotype when challenged with different inocula.

**Fig 3:**
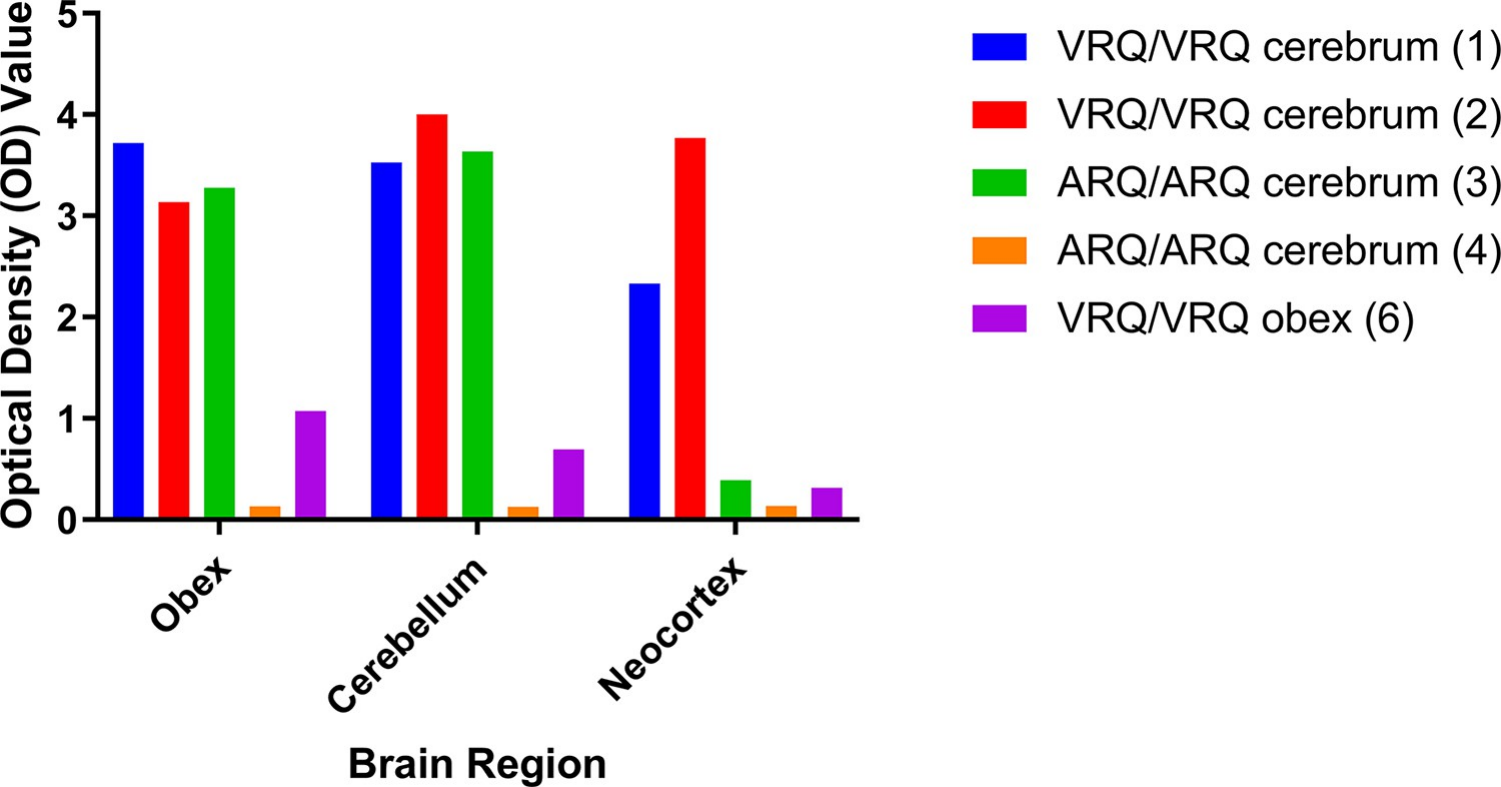
Quantitative analysis of PrP^Sc^ present in brainstem, cerebellum, and neocortex. Differences in the amount of PrP^Sc^ present in brain regions observed by IHC (Fig 2) can be assessed using EIA. The VRQ/VRQ sheep (sheep 1 and 2) have high OD values in all three brain regions. The positive ARQ/ARQ sheep (sheep 3) had high OD values for brainstem and cerebellum but a lower score for the neocortex compared to VRQ sheep that received the same inoculum. The ARQ/ARQ sheep (sheep 4) without immunoreactivity by IHC also was negative by EIA in all three brain regions. A VRQ/VRQ sheep challenged with the scrapie agent from deer brainstem (sheep 6) was positive by EIA but with low OD values relative to sheep challenged with inoculum from deer cerebrum.

To determine if the relative amounts of PrP^Sc^ were consistent with immunohistochemical results we used EIA to quantify PrP^Sc^ in the obex, cerebellum, and neocortex (Fig 3). EIA results for each dilution and brain region are listed in S3 Table. Consistent with IHC immunoreactivity in Fig 2, the optical density values for each brain region were higher in the sheep inoculated with deer cerebrum (sheep 1, 2, and 3) compared to sheep 6 that was challenged with deer brainstem. In addition, VRQ/VRQ sheep (sheep 1 and 2) had higher optical density values compared to ARQ/ARQ sheep (sheep 3) in the neocortex. These results indicate that immunohistochemistry intensity reflects EIA optical density values and abundance of PrP^Sc^ between sheep genotype and inoculum source of the three sheep brain regions tested.

**Fig 4:**
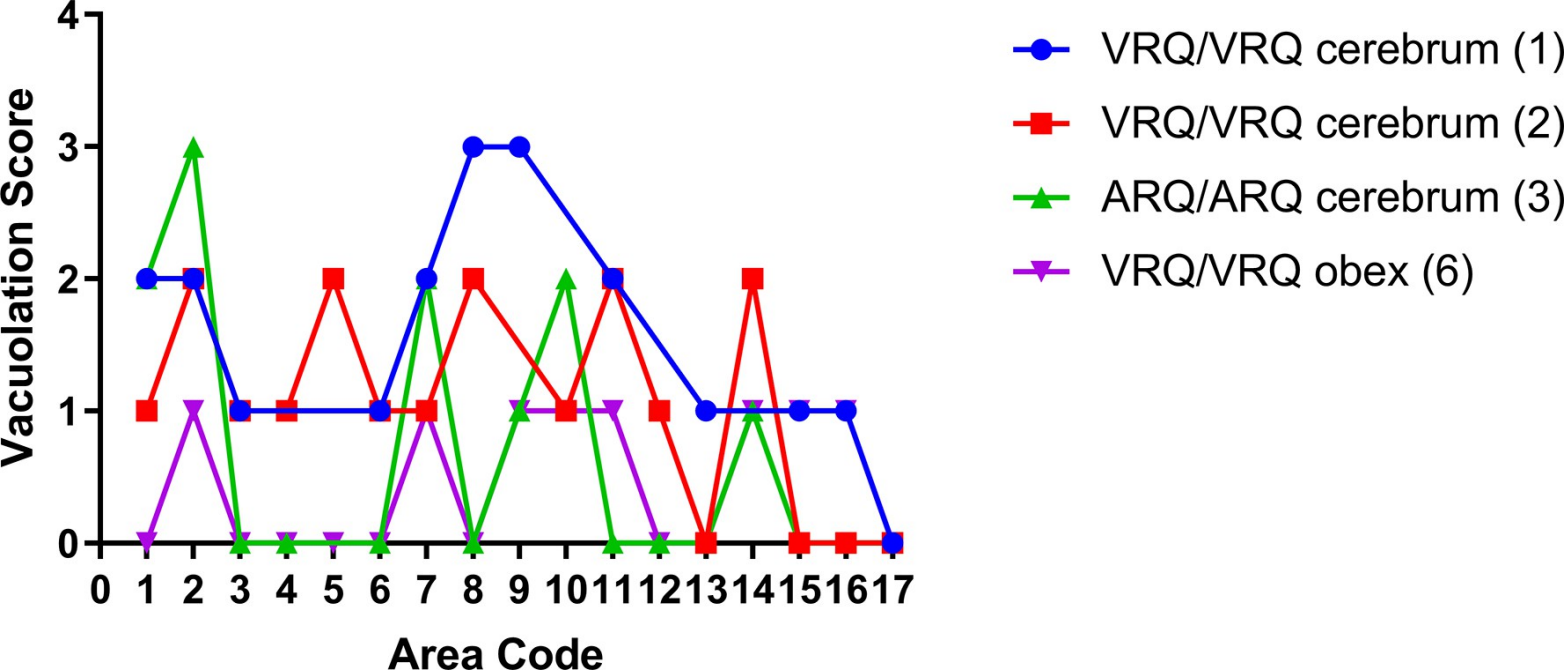
Gray matter vacuolation profiles for sheep inoculated with the WTD scrapie agent. Area codes for specific brain regions are defined in Table 2. Variations in vacuolation scores between sheep of different genotypes challenged with same inoculum and between sheep of the same genotype challenged with different inocula. Similar vacuolation scores were seen in VRQ/VRQ sheep that received WTD cerebrum (sheep 1 and 2). ARQ/ARQ sheep (sheep 3) and the VRQ/VRQ sheep (sheep 6) had lower vacuolation scores compared to the VRQ/VRQ sheep challenged with the WTD cerebrum. Significant differences were seen between sheep 1 and 3 (*) and sheep 1 and 6 (**). Sheep 4 was omitted due to all areas receiving a score of 0 for vacuolation.

Since spongiform change is a hallmark feature of prion disease, we used vacuolation lesion profiling to compare the severity and distribution of disease-associated spongiform change in the brains of sheep of the current study. The results are plotted in Fig 4. Differences in vacuolation scores were found between sheep of different genotypes, for example sheep 1 (VRQ/VRQ) and 3 (ARQ/ARQ) that were both inoculated with the WTD scrapie agent from deer cerebrum. A difference also was found between sheep 1 and 6 that were sheep of the same genotype (VRQ/VRQ) but challenged with inocula derived from different brain regions of the scrapie-affected deer. No spongiform change was observed in sheep 4 (ARQ/ARQ).

**Table 2:** Neuroanatomical areas assessed for gray matter vacuolation scoring.

Neuroanatomical area	Area Code
Nucleus of the solitary tract	1
Nucleus of the spinal tract of V	2
Hypoglossal nucleus	3
Vestibular nuclear complex	4
Cochlear nucleus	5
Cerebellar vermis	6
Central grey matter	7
Rostral colliculus	8
Medial geniculate nucleus	9
Hypothalamus	10
Nucleus dorsomedialis thalami	11
Nucleus ventralis lateralis thalami	12
Frontal cortex	13
Septal nuclei	14
Caudate Nucleus	15
Putamen	16
Claustrum	17

**Fig 5:**
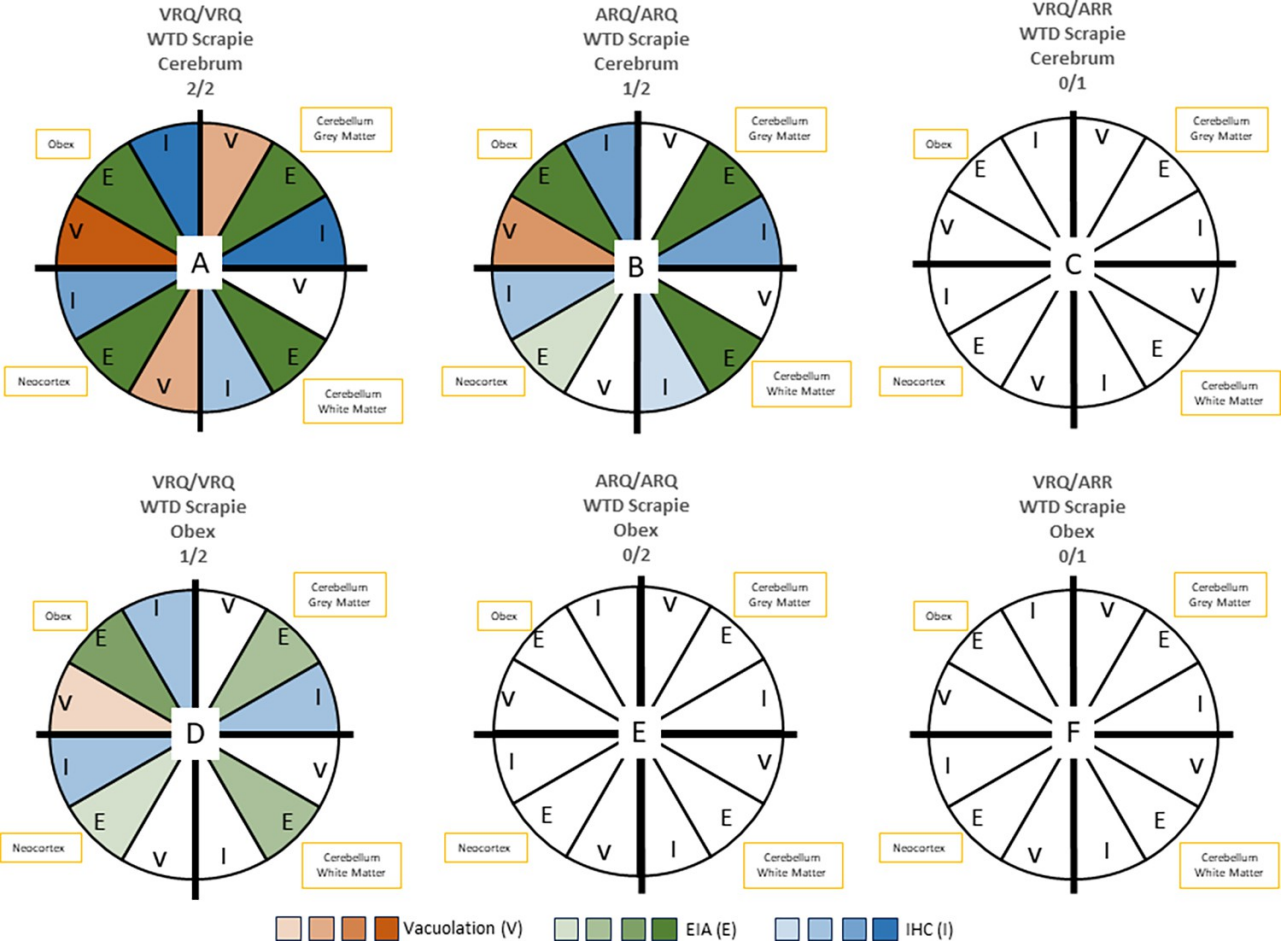
Association of vacuolation, EIA, and immunohistochemistry data with results by sheep genotype and inoculum source. This summary demonstrates differences between sheep of different genotypes challenged with the same inoculum (A-C received inoculum from deer cerebrum; D-F received inoculum from deer obex) and sheep of the same genotype challenged with different inocula. Each quadrant represents a brain region tested (brainstem at the level of the obex, cerebellar grey matter, cerebellar white matter, or neocortex). Each wedge in the quadrant is a different test (vacuolation, EIA, or IHC). The color of the wedge represents the severity of the results from each test, where lower scores received a lighter shade and higher scores received a darker shade. Negative assays are denoted in white. A) VRQ/VRQ with deer cerebrum represents sheep 1 and 2; B) ARQ/ARQ sheep with deer cerebrum represents sheep 3; C) VRQ/ARR with deer cerebrum sheep 5. D) VRQ/VRQ sheep with deer obex represents sheep 6; E) ARQ/ARQ with deer obex represents sheep 8 and 9; F) VRQ/ARR with deer obex represents sheep 10.

Fig 5 summarizes the differences in magnitude between sheep of different genotypes challenged with the same inoculum and sheep of the same genotype challenged with different inocula using three different assays: vacuolation profiling, enzyme immunoassay, and immunohistochemistry.

**Fig 6:**
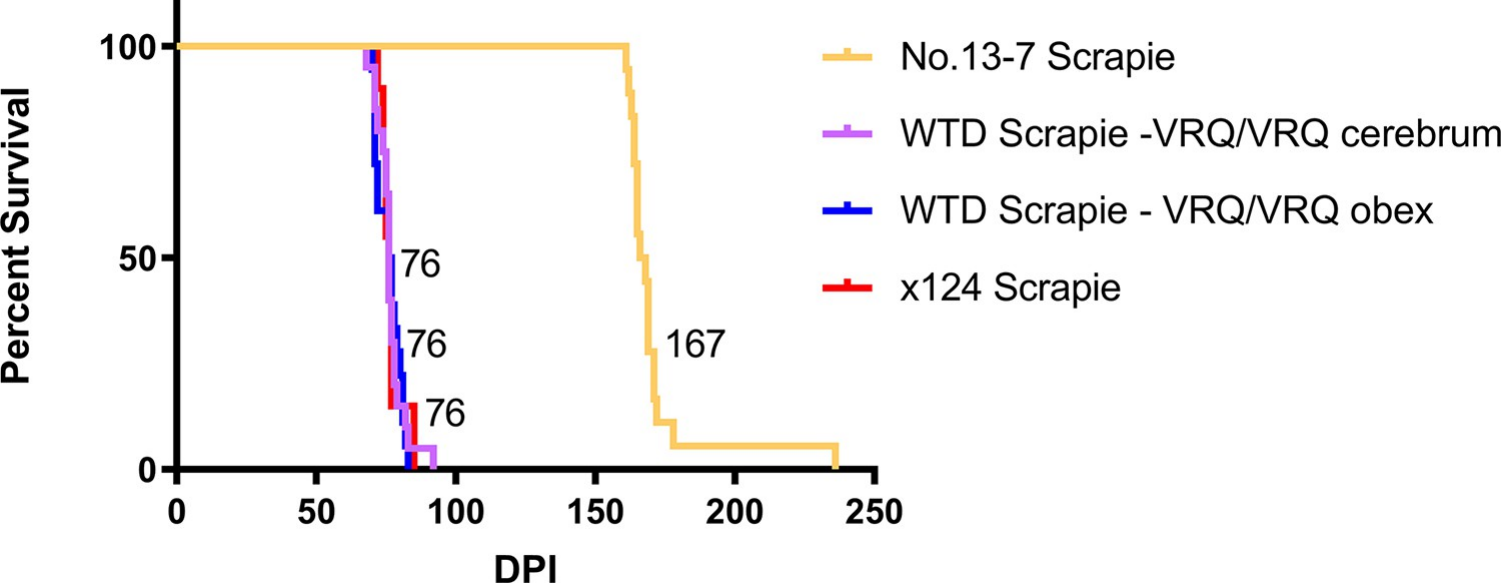
Reduction in No.13-7 incubation period in ovinized mice after passage through deer. Incubation period of No.13-7 inoculum in VRQ/VRQ genotype sheep is reduced by passage through deer. WTD scrapie in sheep has a shorter incubation time in ovinized transgenic mice (Tg338) when compared to the original No.13-7 inoculum and has a similar incubation time of 76 days post-inoculation (DPI) to that of another scrapie strain, x124. All inocula shown are from sheep with the VRQ/VRQ genotype.

Previous studies demonstrate that sheep of the ARQ/ARQ genotype have a faster incubation period when inoculated with the original No.13-7 isolate [6]. In contrast, when sheep were challenged intranasally with the x124 isolate, sheep of the VRQ/VRQ genotype (6.9 MPI) had a fast incubation period, and sheep of the ARQ/ARQ genotype did not develop disease [6]. The current study demonstrates that the WTD scrapie agent had a faster incubation period in VRQ/VRQ sheep (similar to x124 isolate). To investigate the apparent change in phenotype, we used bioassay in transgenic mice (Tg338) that express ovine VRQ *PRNP* to compare attack rates and incubation period between the WTD scrapie inoculum and the original No.13-7 scrapie isolate. All scrapie strains had a 100% attack rate with varying incubation periods. Mice inoculated with the WTD scrapie agent from VRQ/VRQ sheep (inoculum from sheep 2 and 6, Table 1) had a much shorter incubation period (76 +/- 5 DPI; Fig 6) compared to those inoculated with the original No.13-7 scrapie isolate from either a VRQ/VRQ (167 +/-17 DPI; Fig 6) or an ARQ/ARQ (266 +/- 48 DPI) sheep. Mice inoculated with the WTD scrapie agent had a similar incubation period to the x124 isolate from a VRQ/VRQ sheep (76 +/- 4 DPI; Fig 6). Survival analysis of ovinized mice inoculated with the No.13-7, WTD scrapie, and x124 isolates demonstrates that the WTD scrapie agent is significantly shorter than the No.13-7 isolate (Tukey’s multiple comparisons test; p-value <0.0001) and not different from the x124 isolate (Tukey’s multiple comparisons test; p-value >0.9999).

**Fig 7:**
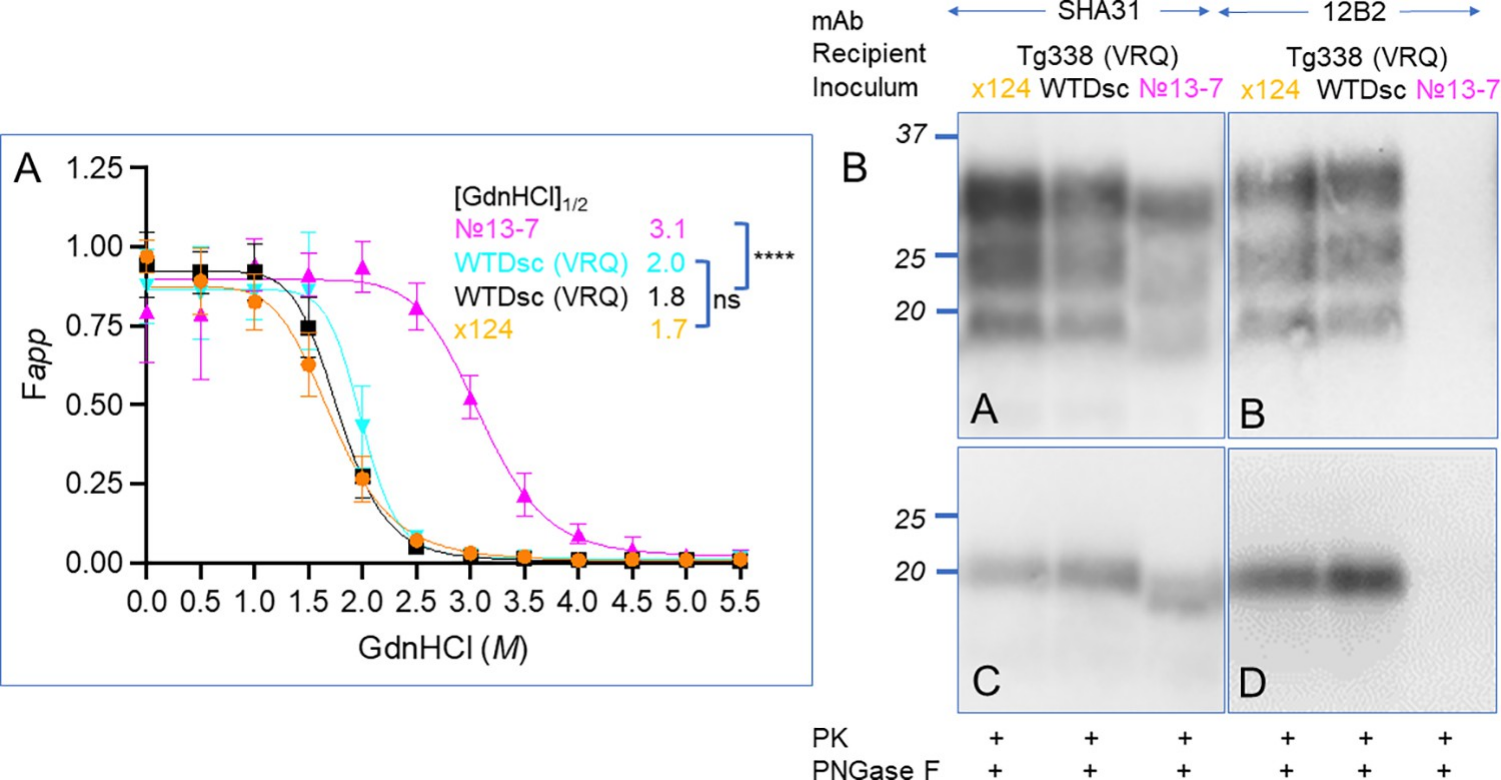
Conformational stability and molecular profiles after passage to an ovinized mouse model support emergence of x124 strain after passage of No. 13–7 strain through white-tailed deer. A) Unfolding curves of were generated from the brains of Tg338 (ovine VRQ *PRNP*) that were inoculated with x124 scrapie from sheep, WTD scrapie after passage through sheep (sheep #2, sheep #6), and the original 13–7 scrapie inoculum. Curves generated from PrP^Sc^ from the brains of mice inoculated with x124 or WTD scrapie passaged through sheep were similar with a [GdnHCl]_1/2_ near 1.8 M, whereas the original scrapie inoculum was significantly more stable with a [GdnHCl]_1/2_ of 3.1 M. B) Western blots of these isolates in Tg338 mice demonstrate a slightly higher apparent molecular mass of the x124 and WTD scrapie isolates than that of the original No. 13–7 inoculum (mAb SHA31). When these tissues are probed with the N-terminal antibody 12B2, binding is present in brain homogenates from mice inoculated with x124 or WTD scrapie passaged through sheep, but not with the No. 13–7 isolate. WTDsc, WTD scrapie agent. Labels in magenta, orange and black indicate brains were used for both western and dot blot assays. [GdnHCl]_1/2_, half-maximal denaturation concentration of PrP^Sc^. F*app*: fraction of apparent PrP^Sc^. ****, p< 0.0001, ns, not statistically significant. Error bars, mean ±SD of data from three mice per group, each mouse brain was analyzed in triplicate.

Conformational stability assays were performed on brain tissues from Tg338 mice inoculated with material from VRQ/VRQ sheep: 1) classical scrapie isolate No.13-7, 2) classical scrapie isolate x124 [6] 3) scrapie agent from WTD cerebrum (sheep 2), or 4) scrapie agent from WTD brainstem (sheep 6). We treated mouse brain homogenates with protein denaturant guanidine hydrochloride (GdnHCl) at concentrations from 0 to 5.5 M with an interval of 0.5 M in 96-well plates, and then digested with proteinase K to remove PrP^C^ and denatured PrP^Sc^. The relative levels (F_APP_) of the undenatured and PK-resistant PrP^Sc^ was determined using a dot-blot assay with SHA31 antibody. The [GdnHCl] _1/2_ values of Tg338 mice inoculated with classic scrapie isolates No.13-7 and x124 are 3.1 M and 1.7 M, respectively. No.13-7 scrapie isolate is ~1.8 times as resistant to GdnHCl denaturation as x124 isolate (Fig 7A). However, after the No.13-7 scrapie isolate was passaged through WTD to sheep and then to Tg338 mice, its conformational stability decreased ~1.8 times, down to 1.8 M and 2.0 M for sheep 2 and 6, respectively. Its denaturation curve was significantly left-shifted and clustered with that of x124 isolate (Fig 7A). The isolates tested in Tg338 mice from the present study have conformation stability curves similar to classical scrapie isolate x124 and have significantly less resistance to denaturation with GdnHCl than the original No. 13–7 scrapie isolate passaged through Tg338 mice (Fig 7A). Furthermore, western blotting demonstrates that the x124 and WTD scrapie agent have molecular profiles with a non-glycosylated band of approximately 20 KDa, but the No.13-7 isolate has a slightly smaller non-glycosylated fragment of approximately 19 KDa (Fig 7B). When these isolates are probed with 12B2, an N-terminal antibody near the PK- cleavage point, the x-124 and WTD scrapie agents demonstrate immunoreactivity, whereas the lower molecular mass No. 13–7 isolate does not.

**Fig 8:**
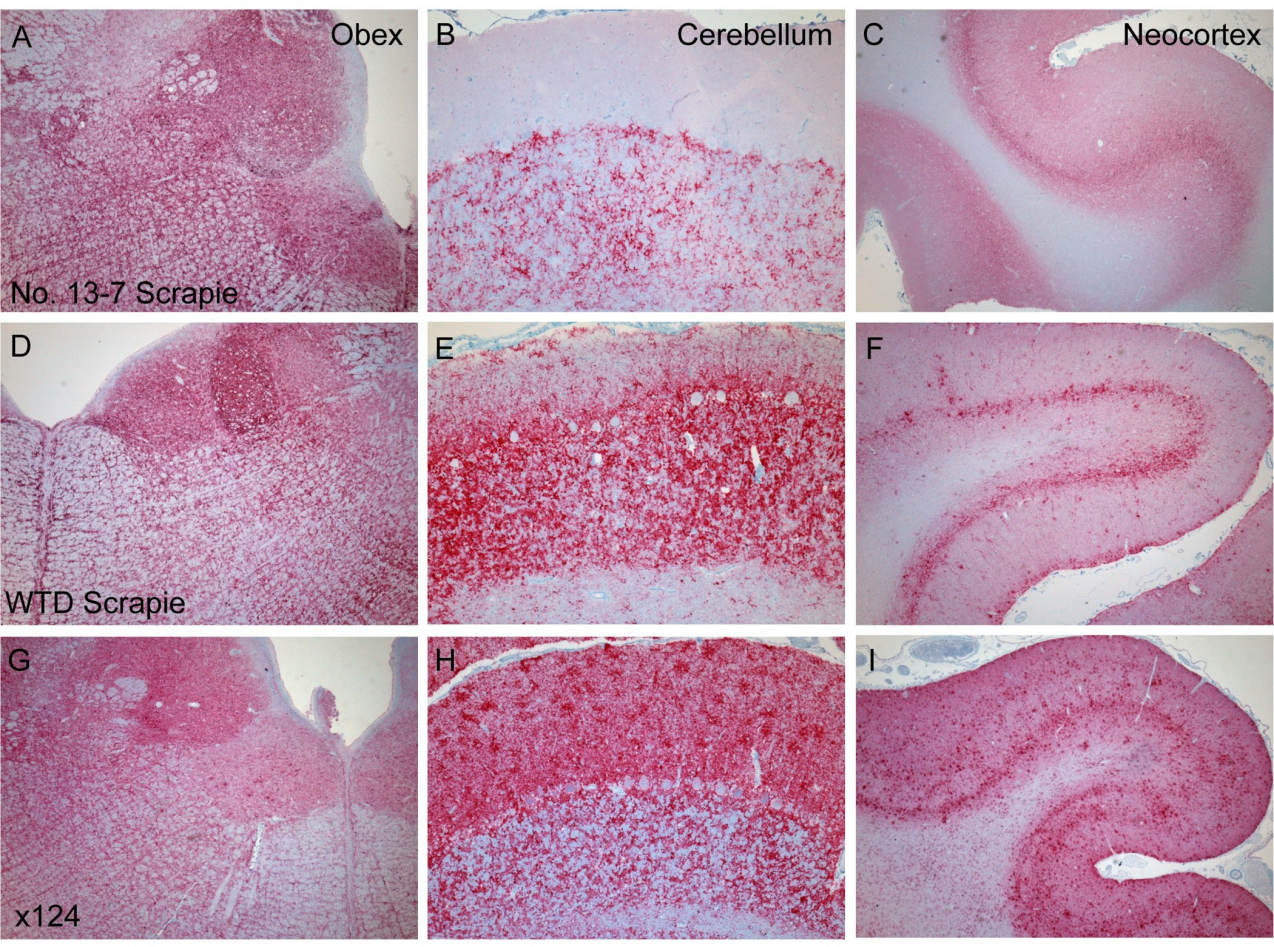
Comparison of PrP^Sc^ accumulation between scrapie strains. PrP^Sc^ distribution differences are noticeable in various brain regions of sheep inoculated with different scrapie isolates. The original No.13-7 scrapie inoculum in sheep is represented in panels A-C. WTD scrapie in sheep, sheep 2, has more PrP^Sc^ labeling in the cerebellum (E) and neocortex (F) compared to No.13-7 (B, C) and also similar, but less, labeling to x124 (G-I).

Since the incubation period in sheep challenged with the agents of WTD scrapie or x124 was faster in VRQ/VRQ sheep compared to ARQ/ARQ sheep [6,10], we used immunohistochemistry to compare PrP^Sc^ accumulation to previously published work in sheep of the VRQ/VRQ genotype challenged with the No.13-7, WTD scrapie, or x124 agents [6]. All inocula had similar widespread accumulation in the obex (Fig 8A, 8D and 8G). Differences in the intensity of immunoreactivity in the cerebellum and neocortex between inocula groups were notable. Sheep inoculated with No.13-7 had less extensive PrP^Sc^ immunoreactivity in the cerebellum compared to the cerebellum of sheep challenged with the WTD scrapie or x124 agents (Fig 8B, 8E and 8H). There was little immunoreactivity in the molecular layer of the cerebella of sheep inoculated with No.13-7, whereas sheep inoculated with either WTD scrapie or x124 had intense, widespread immunoreactivity. There was less PrP^Sc^ deposition in the granular layer of the cerebella in No.13-7 challenged sheep compared to WTD scrapie or x124 challenged sheep. Stellate, small aggregates, linear, and granular labeling are present in neocortical layers I-III and V of WTD scrapie inoculated sheep and not present in No.13-7 inoculated sheep (Fig 8C, 8F and 8I). Granular and linear labeling was not as prominent in layer IV of No.13-7 inoculated sheep compared to WTD scrapie or x124 inoculated sheep. Sheep challenged with WTD scrapie or x124 also had intense immunoreactivity of PrP^Sc^ in layer IV of the neocortex compared to those inoculated with No.13-7 that had mild immunoreactivity in this layer. PrP^Sc^ deposition is also present in neocortical white matter of WTD scrapie or x124 sheep, whereas it is absent in the No.13-7 sheep (Fig 8). These results indicate that there are changes in PrP^Sc^ deposition in the brain after the No.13-7 scrapie isolate has been passaged in deer. Specifically, sheep challenged with the WTD scrapie agent had PrP^Sc^ immunoreactivity patterns more similar to sheep challenged with x124 than sheep challenged with the original No.13-7.

## Discussion

**Fig 9:**
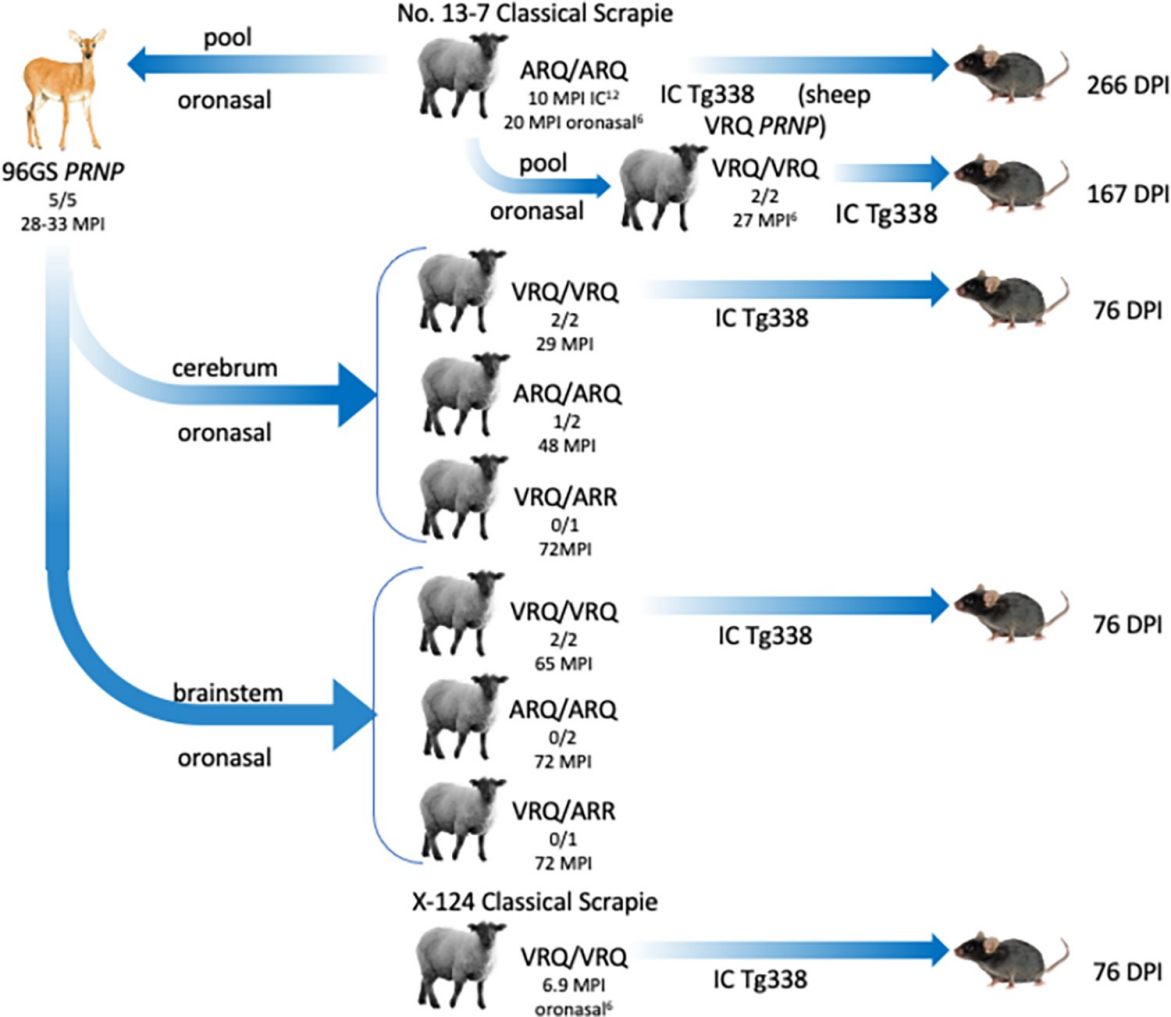
Summary of the No.13-7 scrapie isolate serial passages. The original scrapie isolate was passaged four times by the intracranial route in sheep of the ARQ/ARQ genotype prior to its oronasal challenge in WTD. A cerebrum or brainstem sample from a deer with clinical disease was used to oronasally challenge sheep of various genotypes. The first two sheep to develop disease had the VRQ/VRQ genotype (29 MPI) and were challenged with the scrapie agent from deer cerebrum. One ARQ/ARQ sheep challenged with deer cerebrum also developed disease but at 48 MPI. Sheep challenged with the scrapie agent from deer brainstem tested positive for PrP^Sc^ but with an extended incubation period of 65 MPI. Also shown is the inoculation route from WTD scrapie challenged sheep to Tg338 mice expressing ovine PrP. The mouse passage results can be found in Fig 6 of the current study. The animal figures were created by Corey Summers.

The interspecies transmissions of the No.13-7 scrapie isolate in the current study are shown in Fig 9. The original US No.13-7 scrapie inoculum was serially passaged by the intracranial route in sheep of the ARQ/ARQ genotype [11]. Brain material from two of these sheep was pooled to challenge white-tailed deer by the oronasal route [9]. Two brain regions from these deer (cerebrum and obex) had different molecular weight profiles by western blot [9]. In the current study, samples of brainstem and cerebrum from scrapie-affected deer were used to challenge Suffolk sheep by the oronasal route. To assess strain properties on the same PRNP background, various inocula were compared in Tg338 mice that express the sheep VRQ prion protein.

### Sheep are susceptible to the scrapie agent derived from white-tailed deer

In the present study, the first sheep to develop disease were challenged with the scrapie agent derived from deer cerebrum. In this challenge group, sheep of the VRQ/VRQ genotype developed disease faster than sheep of the ARQ/ARQ genotype. Only one of the five sheep challenged with the scrapie agent derived from deer brainstem developed clinical signs, and that sheep was of the VRQ/VRQ genotype. Overall, only one of the four sheep with the ARQ/ARQ genotype developed disease. This contrasts with previous studies where all sheep developed clinical signs after inoculation with the US No.13-7 sheep scrapie isolate. When inoculated with the No. 13–7 agent from sheep, sheep of the ARQ/ARQ genotype developed disease faster than sheep of the VRQ/VRQ genotype [6]. In the present study, passage of the US No.13-7 scrapie isolate through white-tailed deer results in sheep of the VRQ/VRQ genotype developing disease faster than sheep of the ARQ/ARQ genotype, which supports a switch in phenotypic features.

Sheep of the ARQ/ARR genotype challenged with either cerebrum or brainstem from deer failed to develop disease. This is concordance with our previous work where sheep of the ARQ/ARR genotype did not develop disease or accumulate PrP^Sc^ when challenged orally with the No. 13–7 scrapie isolate [12,13], and is consistent with the presence of arginine (R) at codon 171 providing more resistance to disease [5].

We have shown that the transmission of the scrapie agent derived from white-tailed deer to sheep is possible by the oronasal route. In addition, passage of the original scrapie isolate through white-tailed deer results in a disease phenotype switch. The US No.13-7 scrapie isolate had a more rapid incubation period in sheep of the A ARQ/ARQ genotype prior to passage in deer and a shorter incubation period in sheep of the VRQ/VRQ genotype after passage through deer similar to the x-124 scrapie isolate [6,10].

### Change in disease presentation after passage through deer

Previously, two different western blot patterns were observed in the brains of deer challenged with the sheep scrapie isolate, No.13-7; cerebrum had a scrapie-like pattern while brainstem at the level of the obex had a CWD-like pattern [8,9]. Brains from sheep of the current study that were positive for PrP^Sc^ had a western blot molecular mass profile that looked similar to the original sheep scrapie isolate. This profile was consistent among sheep regardless of sheep genotype and inoculum (cerebrum or obex) source they were challenged with. This finding is in contrast with another report of experimental intracranial transmission of elk CWD prions to sheep that found that the molecular mass of the unglycosylated band of sheep-passaged elk CWD was not similar to naturally acquired sheep with scrapie or elk with CWD [14]. In the sheep reported here, a single western blot profile results after passage through deer, that is indiscernible from the original No.13-7 scrapie inoculum. These results demonstrate that passage of the original No.13-7 scrapie isolate through deer does not influence the western blot molecular mass profile in sheep. However, differences in immunohistochemistry profiles and incubation period between sheep in the current study inoculated with deer cerebrum or brainstem (see below) as compared to No. 13–7 inoculated sheep are in support of a phenotype switch.

Differences in immunoreactivity profiles were observed between the brains of sheep of different genotypes challenged with the same inoculum and sheep of the same genotype challenged with different inocula. When comparing immunohistochemistry patterns between samples from sheep challenged with deer cerebrum, sheep of the VRQ/VRQ genotype had more PrP^Sc^ immunoreactivity in the cerebellum and neocortex compared to sheep of the ARQ/ARQ genotype. However, previous work in sheep challenged with the original No.13-7 isolate described much less extensive immunoreactivity in the neocortex of sheep with the VRQ/VRQ genotype in comparison to sheep of the ARQ/ARQ genotype [6]. This is consistent with immunoreactivity differences described in naturally and experimentally challenged scrapie cases. PrP^Sc^ deposition assessed by immunohistochemistry was different between sheep of the ARQ/ARQ sheep and VRQ/VRQ genotype in cases of natural scrapie [15–17]. Sheep with the ARQ/ARQ genotype had predominately punctate patterning in the dorsal motor nucleus of the vagus nerve (DMNV), whereas sheep of the VRQ/VRQ genotype had predominately granular and coalescing patterning in the DMNV [15]. When comparing Welsh Mountain VRQ/VRQ sheep to Suffolk ARQ/ARQ sheep with naturally acquired scrapie, sheep of the VRQ/VRQ genotype had more vascular plaque PrP^Sc^ accumulation throughout the brain, whereas sheep of the ARQ/ARQ genotype had more astrocyte-associated and neuropil PrP^Sc^ accumulation [16]. Differences in immunohistochemistry PrP^Sc^ patterns also were found in sheep orally challenged with the scrapie agent. Sheep of the VRQ/VRQ genotype had prominent neuronal and glial intracellular aggregates, whereas sheep of the ARQ/ARQ genotype presented with more stellate and astrocyte-associated types and no glia-associated aggregates [17]. Consistent with these findings, the present study demonstrates that experimental transmission by the oronasal route can result in immunoreactivity differences between sheep genotypes.

Differences in the amount of PrP^Sc^ immunoreactivity were observed in sheep of the same genotype when challenged with different inocula sources. Sheep with the VRQ/VRQ genotype challenged with the scrapie agent derived from deer cerebrum had extensive PrP^Sc^ in the brainstem, cerebellum, and neocortex. In contrast, sheep (VRQ/VRQ) challenged with the scrapie agent from deer brainstem had less immunoreactivity in these areas. We also noted differences in immunoreactivity in brain regions of sheep of the VRQ/VRQ genotype when challenged with two different inocula, No.13-7 from a pool of ARQ/ARQ sheep and x124 from a single VRQ/ARR sheep [6]. The differences in immunoreactivity between sheep genotypes and inocula source supports our conclusion that a change in phenotype occurs after the original No. 13–7 scrapie isolate has been passaged in deer.

### Passage of no.13-7 through white-tailed deer results in x124 phenotype

Passage of the No.13-7 isolate through deer and back to sheep resulted in a shortened incubation period in the ovinized mouse line, Tg338. This shorter incubation period was similar to another US scrapie isolate, x124, originally isolated from the western US. Conformational stability of each of these isolates was compared using GdnHCl denaturation. In the present study, the x124 isolate and deer scrapie isolates unfolded at much lower concentrations of denaturant than the No. 13–7 inoculum further supporting a phenotype switch. The more rapid incubation and greater susceptibility to denaturant corroborates previous work by others and suggests that less stable prions replicate more rapidly than stable prions [18,19]. In addition, western blotting of mice inoculated with the x124 and deer scrapie isolates have a similar profile with non-glycosylated band at approximately 20 kDa, which is higher than the original No. 13–7 scrapie. Probing these isolates with the N-terminal antibody 12B2 further differentiates these isolates since the No. 13–7 isolate with the lower apparent molecular mass does not demonstrate immunoreactivity.

The bioassay findings led us to compare PrP^Sc^ immunoreactivity between VRQ/VRQ sheep challenged with the No.13-7, WTD scrapie, or x124 agents. In contrast to sheep inoculated with No.13-7, we found that sheep inoculated with the WTD scrapie agent had more PrP^Sc^ immunoreactivity in the brainstem, cerebellum, and neocortex. Similar immunoreactivity patterns were observed in the brains of sheep inoculated with WTD scrapie and x124. We previously described differences in PrP^Sc^ immunoreactivity patterns in VRQ/VRQ sheep challenged with x124 or No.13-7 scrapie isolates [6].

No. 13–7 classical scrapie prions readily transmit to white-tailed deer. The results of the current study indicate that on return passage to sheep there is a change in disease phenotype. In sheep, the relationship between incubation period and genotype is reversed; the original No.13-7 scrapie inoculum produces a shorter incubation period in ARQ/ARQ sheep compared to VRQ/VRQ sheep, while the deer-passaged scrapie agent results in a shorter incubation period in VRQ/VRQ sheep. In addition, passage of the No.13-7 isolate through deer results in a change in the pattern of PrP^Sc^ deposition in the brain of affected sheep such that the PrP^Sc^ patterns in VRQ/VRQ sheep challenged with WTD scrapie are similar to classical scrapie strain x124. Bioassay and conformational stability assays further support emergence of x124-like properties after passage of No. 13–7 through deer. We are unsure if using SS96 rather than wild-type (GG96) deer for the original passage of the scrapie agent played a role in the phenotype switch observed. Since expression S96 prion protein has been associated with prolonged incubation periods [20] and decreased risk of developing CWD [21], selection for the S96 codon is being actively pursued in farmed cervids. It is possible that selection of deer for CWD prevention may increase the chances of transmission of the scrapie agent to deer if a deer were to be exposed. In summary, this work demonstrates that interspecies transmission of prion isolates can result in the emergence of new strain properties that could alter the host range or require different management strategies to control disease spread.

## Methods and materials

### Ethics statement

All animal experiments described were reviewed and approved by the National Animal Disease Center’s Institutional Animal Care and Use Committee (protocol number 2777 [sheep] and 2730 [mouse bioassay]) and were carried out in strict accordance with the *Guide for the Care and Use of Laboratory Animals* (Institute of Laboratory Animal Resources, National Academy of Sciences, Washington DC) and the *Guide for the Care and Use of Agricultural Animals in Research and Teaching* (Federation of Animal Science Societies, Champaign, IL).

### Sheep experiment

Suffolk lambs from the National Animal Disease Center’s (NADC) scrapie-free flock of *PRNP* genotype VV_136_RR_154_QQ_171_ (VRQ/VRQ; n = 4), AV_136_RR_154_QR_171_ (VRQ/ARR; n = 2), and AA_136_RR_154_QQ_171_ (ARQ/ARQ; n = 4) were challenged by the oronasal route at approximately 5 months of age with a 10% brain homogenate from No.13-7 scrapie-affected WTD cerebrum (n = 5) or brainstem (n = 5). Sheep were observed daily throughout the duration of the experiment. Upon development of intercurrent disease or neurologic signs consistent with TSE, or at the end of the experiment (72 months post-inoculation), sheep were euthanized and necropsied.

### Classical scrapie inoculum

The inoculum was composed of brain homogenate from a white-tailed deer (#5) that was challenged oronasally with the scrapie isolate No.13-7 [9]. The deer received scrapie inoculum (No.13-7) that was pooled from two ARQ/ARQ sheep after serial intracranial passage in sheep of the ARQ/ARQ genotype [11]. A 10% w/v brain homogenate from either the deer cerebrum or brainstem at the level of the obex was made in phosphate buffered saline (PBS) with 100 μg/mL gentamicin. The high (brainstem) and low (cerebrum) migration patterns of the two inocula sources were confirmed by western blot. The relative amounts of PrP^Sc^ of the two inocula sources were also quantified by enzyme immunoassay (EIA) (see below). EIAs were done on samples from both brain regions at dilutions of 1:1, 1:25, 1:50, and 1:100. Optical density (OD) values are listed in S1 Table. One mL of 10% w/v homogenate made from either the deer cerebrum or brainstem was oronasally inoculated into the left nostril of lambs as previously described [6].

### Sample collection

A complete necropsy was performed on all sheep. Two sets of tissue samples were taken, one was frozen and the second was fixed in 10% buffered formalin, paraffin embedded, and sectioned at 5 μm for staining with hematoxylin and eosin (HE) or with anti-prion protein antibodies. Tissue samples collected included brain, spinal cord (cervical, thoracic, lumbar), dorsal root ganglion, nerves (optic, sciatic, trigeminal), eye, pituitary, muscle (tongue, masseter, diaphragm, heart), liver, lung, trachea, kidney, spleen, pancreas, adrenal, salivary gland, skin, urinary bladder, intestines (ileum, jejunum, cecum), forestomachs, lymph nodes (retropharyngeal, mesenteric, prescapular, popliteal), tonsils (palatine and nasopharyngeal), thymus, thyroid, and rectal mucosa [6].

### Immunohistochemistry

Paraffin embedded sheep tissues were sectioned and stained for PrP^Sc^ by an automated method using a cocktail of two anti-prion protein monoclonal antibodies, F99/97.6.1 [22] and F89/160.1.5 [23] at a concentration of 5 μg/ml as previously described [6]. Briefly, after deparaffinization and rehydration, tissue sections were autoclaved for 30 min in an antigen retrieval solution (DAKO Target Retrieval Solution, DAKO Corp., Carpinteria, CA, USA) and stained with an indirect, biotin-free staining system containing an alkaline phosphatase labeled secondary antibody (ultraview Universal Alkaline Phosphatase Red Detection Kit, Roche Diagnostics Corporation, Indianapolis, IN, USA) designed for an automated immunostainer (NexES IHC module, Ventana Medical Systems, Inc., Tucson, AZ, USA). The morphology of the PrP^Sc^ deposits was defined as previous [17]. The stellate pattern is characterized by glial-type nuclei with radiating branches of immunoreactivity. The term “aggregates” was used instead of “coalescing” and is defined as amyloid-like deposits that result in the merging of coarse particulate PrP^Sc^ deposits [24]. The linear pattern is observed in the neuropil and has thread-like PrP^Sc^ deposits in a linear form [17]. The term “granular” was used instead of the term “fine punctate” [17]. Granular patterning is the small, diffuse PrP^Sc^ deposits typically found in the neuropil [24,25]. After processing, images were captured using a Nikon DS camera on a Nikon Eclipse 55*i* microscope.

### Western blotting

Frozen samples taken at necropsy were used for immunodetection of the abnormal prion protein by western blot. Western blotting of CNS PrP^Sc^ was conducted and analyzed as previously described [26], protein concentrations of brain homogenates (20 w/v) were determined using bicinchoninic acid assay (BCA) (Piece Biotechnology, Rockford, lL, USA). Homogenates were treated with 50 μg/ml proteinase K (PK) (Roche, Mannheim, Germany) in the presence of 2% sarkosyl for 1 h at 37°C with gentle agitation (500 RPM). PK digestion was terminated with PMSF at a final concentration of 2 mM for 20 min at room temperature. For further deglycosylation analysis, PK-treated and PK-untreated samples were treated with PNGase F (New England Biolabs, Cat. P0704S, Ipswich, MA) at the concentration of 25 units/ug protein for 2.5 hr at 37°C with gentle agitation. PK and/or PNGase F treated and untreated samples were denatured in XT-sample buffer (Bio-Rad Laboratories, Hercules, CA) for 10 min at 95°C and were loaded onto 12% Bis-Tris SDS-PAGE gels (Bio-Rad Laboratories, Hercules, CA), and then proteins were transferred overnight to PVDF-FL membranes (Millipore, Billerica, MA, USA). Membranes were blocked for 1 hour in 5% non-fat milk in TBS-T (1 x Tris-buffered saline and 0.05% Tween 20), probed with SHA31 (Bertin Technologies, Cat. A03213, France) and 12B2 (Wageningen University and Research, Netherlands) mAbs, followed by HRP-conjugated anti-mouse IgG secondary antibody (Millipore-Sigma, Cat. NXA931, Burlington, MA). Membranes were developed using ECL Plus substrate (Thermo Scientific, USA), and imaged with iBright FL-1500 imaging system (ThermoFisher Scientific, Waltham, MA).

### Enzyme immunoassay

The BSE-Scrapie Antigen Test Kit, EIA (IDEXX, Westbrook, ME) was used on different tissues including the brainstem at the level of the obex, cerebellum, and neocortex prepared as 20% homogenates in PBS for the sheep of the current study. The test kit was also used on the inocula (10% homogenate) used to challenge the sheep (S1 Table). The inocula (1% homogenate) used for the mouse bioassay studies were also tested for the relative amount of PrP^Sc^ present (S2 Table). Each sample of the 10% sheep inoculum, 1% mouse inoculum or the 20% homogenate of the three brain regions listed above (S3 Table were first tested for PrP^Sc^ without being diluted (1,1). Samples were then diluted 1:25, 1:50, and 1:100 in PBS to determine the relative amounts of PrP^Sc^. Diluted samples were then assayed on the antigen capture plate with the provided controls using the suggested short protocol with slight modifications. Each 100 μL sample received 25 μL of working plate diluent prior to being added to the plate. The capture plate then incubated for an hour and a half with agitation. After washing, the small ruminant brain conjugate concentrate (SRB-CC) was used as described and incubated for one hour without agitation. The antigen capture plate was then read on a SpectraMax 190 (Molecular Devices, Sunnyvale, CA) with an optical density of 450nm and a reference wavelength of 650nm. The negative sample cut-off value was determined by adding 0.180 to the negative control sample provided in the kit, as described in the protocol. Samples were deemed positive if their OD value was greater than the cut-off value. Since a maximum OD reading of 4.0 using EIA can result from increasing levels of misfolded protein, we ran various dilutions to determine the relative amount of PrP^Sc^ present in each sample.

### Vacuolation profiling

Vacuolation profiles were generated by scoring defined brain (Table 2) regions using hematoxylin and eosin-stained slides. Grey matter scores were based on previous work [27] and are as followed: 0 = no vacuolation, 1 = occasional vacuole, 2 = several vacuoles, evenly distributed, 3 = moderate to many vacuoles evenly distributed, and 4 = severe vacuolation with possible coalescence. Each score for the perspective area was graphed for the corresponding animal, with the exception of sheep 4 that received a score of 0 for each area. An average score for each area for all the sheep was calculated and graphed. The graph was made using GraphPad Prism 7 (GraphPad Software, Inc., La Jolla, CA) and analysis was done using one way ANOVA.

### Mouse bioassay

Brain material was bioassayed in transgenic mice expressing the ovine *PRNP* Tg338 (ovine, PrP^VRQ^) [28]. Five groups of mice were inoculated with brain homogenate prepared from the brainstem at the level of the obex. Inocula were derived from sheep 2 and 6 of the current study, No.13-7 inoculum from and ARQ/ARQ pool (see classical scrapie inoculum above) or a single VRQ/VRQ sheep, and a second scrapie agent from a VRQ/VRQ sheep, x124 [6] (see Fig 9). EIA’s were done on the inoculum sources to determine the relative amounts of PrP^Sc^ as described above. OD values for each inoculum source are listed in S2 Table. Mice (n = 17 for No.13-7, n = 20 for sheep 2, n = 18 for sheep 6, and n = 20 for x124) were intracranially inoculated using 20 μL of a 1% w/v brain homogenate made from sheep. Animal care staff monitored the mice daily for development of clinical signs. Mice were humanely euthanized when clinical signs of poor coordination, ataxia, difficulty/inability to move, and/or poor grooming with urine-stained fur became apparent. Brains from the mice were prepared for EIA by making a 20% homogenate in PBS and performed as described above. Attack rates were determined by taking the number of mice with a positive EIA result divided by the total number of mice. Incubation periods were determined by taking the average days post-inoculation (DPI) of all positive mice per group. Survival analysis was done using GraphPad Prism 7 (GraphPad Software Inc.).

### Conformational stability assays

Conformational stability assays were conducted as previously described [26,29–31]. Briefly, mouse brain homogenates (15 μl, 1%) were denatured in 0–5.5 M GdnHCl (Sigma, G7294) in 96-well plates for 1 h at room temperature. Samples were transferred onto Amersham Protran nitrocellulose membrane (Cytiva) using a Bio-Dot Microfiltration (Bio-Rad). After two PBS washes, the membrane was air-dried for 1 hour, then incubated with 5 μg/ml PK in cell lysis buffer (50 mM Tris-HCl, pH 8.0, 150 mM NaCl, 0.5% sodium deoxycholate, 0.5% Igepal CA-630) for 1 hour at 37°C. PK was inactivated with 2 mM PMSF. Membranes were denatured in 3 M guanidine thiocyanate in Tris-HCl, pH 7.8 for 10 minutes at room temperature. After four washes with PBS, the membrane was blocked with 5% nonfat milk in TBST for 1 hour and probed overnight at 4°C with SHA31 (Cayman Chemical) at a dilution of 1∶5000, followed by HRP-conjugated goat anti-mouse IgG secondary antibody. The membrane was developed with ECL Plus and scanned with ChemiDoc imager (Bio-Rad), and signals analyzed with AzureSpot Pro analysis software (Azure Biosystems). Three biological replicates were analyzed, and each of these biological replicates was analyzed in triplicate. Statistical analysis was performed using Graphpad Prism software (San Diego, CA), statistical significance of [GdnHCl]1/2 was assessed using Student t test.

## Supporting information

S1 Table WTD scrapie inocula EIA data. Deer cerebrum and deer obex inocula samples (10% homogenate at 1:1) were ran on EIA (BSE-Scrapie Antigen Test Kit, EIA, IDEXX, Westbrook, ME) in the following increasing dilutions to determine the amount of PrP^Sc^ in each sample.
(DOCX)

S2 Table EIA data for mouse bioassay inocula. Sheep inoculated with No.13-7, WTD scrapie sheep 2 (VRQ/VRQ sheep inoculated with deer cerebrum), x124, and WTD scrapie sheep 6 (VRQ/VRQ sheep inoculated with deer obex) used as inoculum source (1% homogenate at 1:1) for mouse bioassay were tested via EIA (BSE-Scrapie Antigen Test Kit, EIA, IDEXX, Westbrook, ME) in increasing dilutions to determine the amount of PrP^Sc^.
(DOCX)

S3 Table EIA optical densities of brain regions of sheep infected with the WTD scrapie agent. Samples from the brainstem at the level of the obex, cerebellum, and neocortex (10% homogenate at 1:1) of the animals tested for PrP^Sc^ immunoreactivity in Fig 2 were quantified using EIA (BSE-Scrapie Antigen Test Kit, EIA, IDEXX, Westbrook, ME) in increasing dilutions to determine the relative amount of PrP^Sc^ present in each brain region. The negative sample cut-off value of 0.201 was determined by adding 0.180 to the negative control sample provided in the kit.
(DOCX)
