# Supplementary material for: Disease phenotype of classical sheep scrapie is changed upon experimental passage through white-tailed deer
Source: PLoS Pathog. 2023 Dec 4;19(12):e1011815. doi: 10.1371/journal.ppat.1011815 (PMC10721168; doi:10.1371/journal.ppat.1011815)
Supplement: S1 Table — Deer cerebrum and deer obex inocula samples (10% homogenate at 1:1) were ran on EIA (BSE-Scrapie Antigen Test Kit, EIA, IDEXX, Westbrook, ME) in the following increasing dilutions to determine the amount of PrPSc in each sample. (DOCX) [file ppat.1011815.s001.docx]

**S1 Table. WTD scrapie inocula EIA data**

| Dilution | Deer Cerebrum | Deer Obex |
| --- | --- | --- |
| 1:1 | 4 | 4 |
| 1:25 | 0.999 | 4 |
| 1:50 | 0.611 | 2.46 |
| 1:100 | 0.399 | 1.925 |

Deer cerebrum and deer obex inocula samples (10% homogenate at 1:1) were ran on EIA (BSE-Scrapie Antigen Test Kit, EIA ,IDEXX, Westbrook, ME) in the following increasing dilutions to determine the amount of PrP^Sc^ in each sample.
