# Supplementary material for: Disease phenotype of classical sheep scrapie is changed upon experimental passage through white-tailed deer
Source: PLoS Pathog. 2023 Dec 4;19(12):e1011815. doi: 10.1371/journal.ppat.1011815 (PMC10721168; doi:10.1371/journal.ppat.1011815)
Supplement: S2 Table — Sheep inoculated with No.13-7, WTD scrapie sheep 2 (VRQ/VRQ sheep inoculated with deer cerebrum), x124, and WTD scrapie sheep 6 (VRQ/VRQ sheep inoculated with deer obex) used as inoculum source (1% homogenate at 1:1) for mouse bioassay were tested via EIA (BSE-Scrapie Antigen Test Kit, EIA, IDEXX, Westbrook, ME) in increasing dilutions to determine the amount of PrPSc. (DOCX) [file ppat.1011815.s002.docx]

**S2 Table. EIA data for mouse bioassay inocula**

| Dilution | No.13-7 | Sheep 2 | x124 | Sheep 6 |
| --- | --- | --- | --- | --- |
| 1:1 | 4 | 3.926 | 4 | 2.006 |
| 1:25 | 3.701 | 3.427 | 3.04 | 0.195 |
| 1:50 | 3.176 | 2.917 | 3.441 | 0.167 |
| 1:100 | 2.156 | 1.854 | 2.354 | 0.125 |

Sheep inoculated with No.13-7, WTD scrapie sheep 2 (VRQ/VRQ sheep inoculated with deer cerebrum), x124, and WTD scrapie sheep 6 (VRQ/VRQ sheep inoculated with deer obex) used as inoculum source (1% homogenate at 1:1) for mouse bioassay were tested via EIA (BSE-Scrapie Antigen Test Kit, EIA ,IDEXX, Westbrook, ME) in increasing dilutions to determine the amount of PrP^Sc^.
