# Supplementary material for: Disease phenotype of classical sheep scrapie is changed upon experimental passage through white-tailed deer
Source: PLoS Pathog. 2023 Dec 4;19(12):e1011815. doi: 10.1371/journal.ppat.1011815 (PMC10721168; doi:10.1371/journal.ppat.1011815)
Supplement: S3 Table — Samples from the brainstem at the level of the obex, cerebellum, and neocortex (10% homogenate at 1:1) of the animals tested for PrPSc immunoreactivity in Fig 2 were quantified using EIA (BSE-Scrapie Antigen Test Kit, EIA, IDEXX, Westbrook, ME) in increasing dilutions to determine the relative amount of PrPSc present in each brain region. The negative sample cut-off value of 0.201 was determined by adding 0.180 to the negative control sample provided in the kit. (DOCX) [file ppat.1011815.s003.docx]

**S3 Table. EIA optical densities of brain regions of sheep infected with the WTD scrapie agent**

| **Sheep Genotype: VRQ/VRQ** | | | | | | | | | |
| --- | --- | --- | --- | --- | --- | --- | --- | --- | --- |
| **Dilution** | **Sheep 1- cerebrum inoculum** | | | **Sheep 2- cerebrum inoculum** | | | **Sheep 6- obex inoculum** | | |
|  | Obex | Cerebellum | Neocortex | Obex | Cerebellum | Neocortex | Obex | Cerebellum | Neocortex |
| 1:1 | 3.835 | 3.526 | 2.332 | 3.608 | 4 | 3.77 | 4 | 0.692 | 0.314 |
| 1:50 | 3.717 | 1.994 | 0.458 | 3.134 | 2.319 | 2.242 | 1.071 | 0.201 | 0.203 |
| 1:100 | 3.678 | 1.295 | 0.351 | 2.85 | 1.713 | 0.845 | 1.182 | 0.166 | 0.153 |
| **Sheep Genotype: ARQ/ARQ** | | | | | | |  |  |  |
| **Dilution** | **Sheep 3- cerebrum inoculum** | | | **Sheep 4- cerebrum inoculum** | | |  |  |  |
|  | Obex | Cerebellum | Neocortex | Obex | Cerebellum | Neocortex |  |  |  |
| 1:1 | 4 | 3.635 | 0.391 | 0.132 | 0.129 | 0.137 |  |  |  |
| 1:50 | 3.278 | 1.048 | 0.204 | 0.133 | 0.115 | 0.147 |  |  |  |
| 1:100 | 3.151 | 0.486 | 0.175 | 0.172 | 0.122 | 0.116 |  |  |  |

Samples from the brainstem at the level of the obex, cerebellum, and neocortex (10% homogenate at 1:1) of the animals tested for PrP^Sc^ immunoreactivity in Figure 2 were quantified using EIA (BSE-Scrapie Antigen Test Kit, EIA ,IDEXX, Westbrook, ME) in increasing dilutions to determine the relative amount of PrP^Sc^ present in each brain region. The negative sample cut-off value of 0.201 was determined by adding 0.180 to the negative control sample provided in the kit.
